# Serum chloride concentrations and outcomes in adult patients with cirrhosis: a systematic review and meta-analysis

**DOI:** 10.1186/s12882-025-04466-9

**Published:** 2025-09-26

**Authors:** Mehdi Kashani, Lifang Wei, Waryaam Singh, Supawadee Suppadungsuk, Larry J. Prokop, Kianoush B. Kashani, Juan Pablo Domecq Garces

**Affiliations:** 1 Division of Nephrology and Hypertension, Mayo Clinic, Rochester, MN USA; 2 Faculty of Medicine Ramathibodi Hospital, Chakri Naruebodindra Medical Institute, Mahidol University, Samut Prakan, Thailand; 3 Mayo Clinic Libraries, Mayo Clinic, Rochester, MN USA; 4 Division of Critical Care Medicine, Mayo Clinic, Rochester, MN USA; 5 Division of Nephrology and Hypertension, Department of Medicine, Mayo Clinic, Rochester, MN USA; 6 The Third People’s Hospital Affiliated to Fujian University of Traditional Chinese Medicine, Fuzhou, Fujian 350122 China

**Keywords:** Serum chloride, Hypochloremia, Cirrhosis outcomes, Acute kidney injury, Mortality risk

## Abstract

**Purpose:**

Electrolyte disturbances, including hyponatremia, are common in cirrhosis, with hyponatremia already incorporated into the MELD-Na score as a prognostic marker. However, the prognostic role of serum chloride, the main extracellular anion, remains underexplored. Emerging evidence suggests hypochloremia is independently associated with increased mortality and acute kidney injury (AKI) in cirrhotic patients. Proposed mechanisms include dysregulated activation of the renin-angiotensin, vasopressin, and sympathetic nervous systems, leading to renal vasoconstriction and impaired function. This systematic review evaluates the association between serum chloride levels and outcomes, including mortality and KDIGO-defined AKI rates, aiming to enhance understanding and inform management strategies.

**Methods:**

This review followed PRISMA guidelines and a PROSPERO-registered protocol (CRD42024550945). Comprehensive searches of MEDLINE, EMBASE, Cochrane, Scopus, and Web of Science were conducted through June 6, 2024, without language restrictions. Controlled vocabulary and keywords were used to identify relevant studies. Two independent reviewers performed title, abstract, and full-text screening, with disagreements resolved through consensus or third-party arbitration. Inter-rater reliability was assessed using Cohen’s kappa. Data extraction and risk of bias evaluations were performed using the PROBAST tool. Findings were summarized using a PRISMA flowchart.

**Results:**

Five studies (*n* = 3,150) were included, primarily retrospective cohorts, with one prospective study. Hypochloremia was defined as serum chloride < 99 mEq/L in most studies, except one, which used < 107.35 mmol/L. Cirrhosis etiologies included alcohol-related liver disease (40–64%), hepatitis B (7.9–59.9%), hepatitis C (7–8.9%), and non-alcoholic fatty liver disease (7–11.7%), with fewer cases of autoimmune and cryptogenic causes. Comorbidities included diabetes mellitus (21.3%), hypertension (13.4%), and varices (72–87%), with 62–99% having a history of decompensation. Extrahepatic organ failures were prevalent, affecting 79.4% of patients, with 31.6% and 13.4% experiencing two and three organ failures, respectively.Meta-analysis showed hypochloremia was significantly associated with increased mortality (pooled OR: 2.52; 95% CI: 1.88–3.39, *p* < 0.0001). Individual ORs ranged from 2.08 to 17.42, with low to moderate heterogeneity (I² = 36%). Hypochloremia also correlated with elevated creatinine levels and increased AKI prevalence.

**Conclusion:**

Hypochloremia is a strong predictor of mortality and renal dysfunction in cirrhotic patients. Early recognition and management of hypochloremia are critical to improving outcomes in this high-risk population.

**Clinical trial number:**

Not applicable.

## Introduction

Chronic liver disease and cirrhosis lead to pathophysiological changes that predisposes patients to different water, electrolyte, and acid-base alterations [1, 2]. Hyponatremia, found in 40–60% of patients with cirrhosis [2–4], has become a well-established prognostic marker (MELD-Na score) for short-term mortality prediction [5].

In contrast, the prognostic significance of other electrolytes, particularly serum chloride, remains less understood. As the principal anion in extracellular fluid and the second leading contributor to plasma tonicity, chloride plays a crucial role in various physiological processes [6]. While the biological mechanisms linking dyschloremia to adverse events are still being elucidated, evidence suggests potential effects through alteration in serum pH, reduced cardiac contractility, immunosuppression, and activation of neurohormonal systems leading to excessive afferent renal artery vasoconstriction and decreased renal perfusion [7–13].

Recent studies have identified hypochloremia as an independent predictor of mortality in patients with heart failure, chronic kidney disease (CKD), and critical illness [13–18]. Hypochloremia can result from extrarenal chloride losses such as vomiting, burns or gastrointestinal fistulae, or from renal causes such as diuretics, salt losing nephropathy or adrenal insufficiency. It is also associated with hyponatremia in varying extracellular volume states including normal for example SIADH or hypothyroidism, low for example congestive heart failure or cirrhosis, and high for example early hyperglycemia, and with acid base disturbances such as respiratory acidosis and metabolic alkalosis [12, 19, 20]. Hypochloremia in patients with chronic heart failure (HF) and CKD is an independent risk factor for increased mortality [14–18, 21–23].

This systematic review aims to summarize the pertinent evidence on the association between serum chloride concentrations, mortality and acute kidney injury (AKI) in adult patients with cirrhosis.

## Methods

We conducted this systematic review following the Preferred Reporting Item for Systematic Reviews and Meta-Analyses (PRISMA) statement and a pre-established protocol available on the International Prospective Register of Systematic Reviews (PROSPERO ID: CRD42024550945).

### Information sources and search strategies

A comprehensive literature search was conducted across multiple databases, including Ovid MEDLINE^®^, Ovid EMBASE, Ovid Cochrane Central Register of Controlled Trials, Ovid Cochrane Database of Systematic Reviews, Scopus, and Web of Science, covering all languages from their inception to June 6, 2024. The search was designed and executed by an experienced librarian in collaboration with the study’s principal investigator. Controlled vocabulary and supplementary keywords were used to identify studies examining serum chloride concentrations in adult patients with cirrhosis.

### Eligibility criteria and selection process

We included all full-text, peer-reviewed original articles that examined the relationship between serum chloride levels and cirrhosis outcomes. Eligible studies were retrospective or prospective cohort studies. We excluded case reports, case series, non-human studies, reviews, editorials, author responses, letters, comments, book chapters, conference abstracts, and studies focused on pediatric patients. Although our search included articles in all languages, no non-English articles met the inclusion criteria.

### Study selection, data collection, and analysis

After receiving the librarian search output on the Covidence website [24], two independent investigators (MK and WS) reviewed the abstracts and titles identified through the search strategy. They then evaluated the full-text reports for eligibility. Disagreements were resolved through consensus and arbitration by a third reviewer (KK). The inter-rater agreement between reviewers was measured using Cohen’s kappa (κ) coefficient. The PRISMA flowchart was used to represent the study selection process. Two authors (NN and SR) extracted data, including the first author’s name, the publication year, database, definition of sepsis, number of included patients and patient characteristics (e.g., sex, age), outcome (e.g., type and incidence of mortality and AKI), time of data collection.

### Quality assessment

Two authors (NN and HHT) evaluated the risk of bias in the selected articles using the Risk of Bias in Non-Randomized Studies of Interventions (ROBINS-I) tool [25]. This tool assesses key domains, including confounding, selection of participants, classification of interventions, deviations from intended interventions, missing data, measurement of outcomes, and selection of reported results. The evaluation categorized the risk of bias as “low,” “moderate,” or “high” based concerns within each domain (Table 1).

**Table 1:**
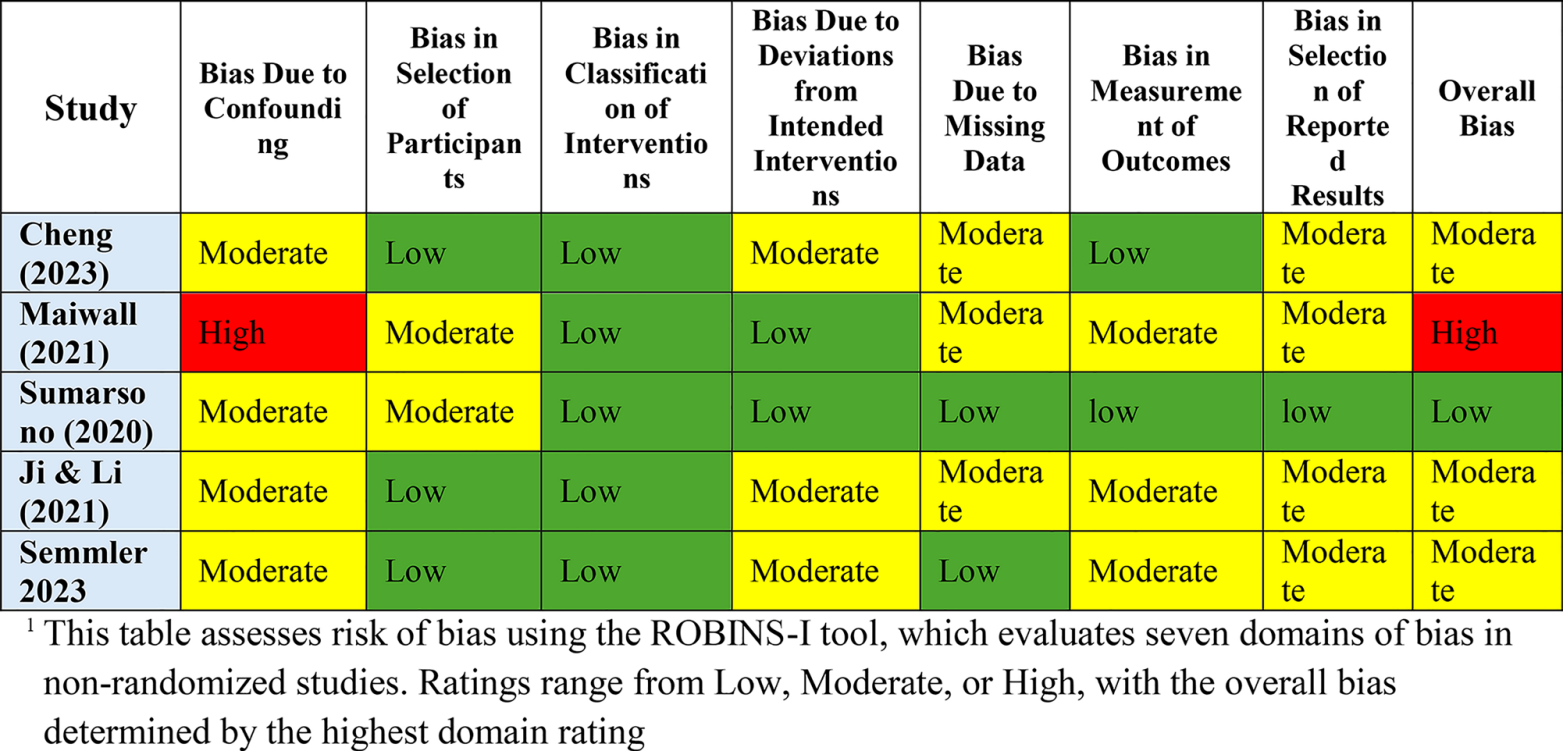
Risk of bias assessment using the ROBINS-I tool across included studies^1^

The overall risk of bias for the included studies was determined using the ROBINS-I tool, ensuring reliability and consistency in methodological quality assessment. Studies with moderate bias often demonstrated concerns related to confounding and outcome measurement, whereas studies with low bias exhibited minimal issues, reflecting stronger methodological integrity.

## Result

### Study selection

**Fig. 1:**
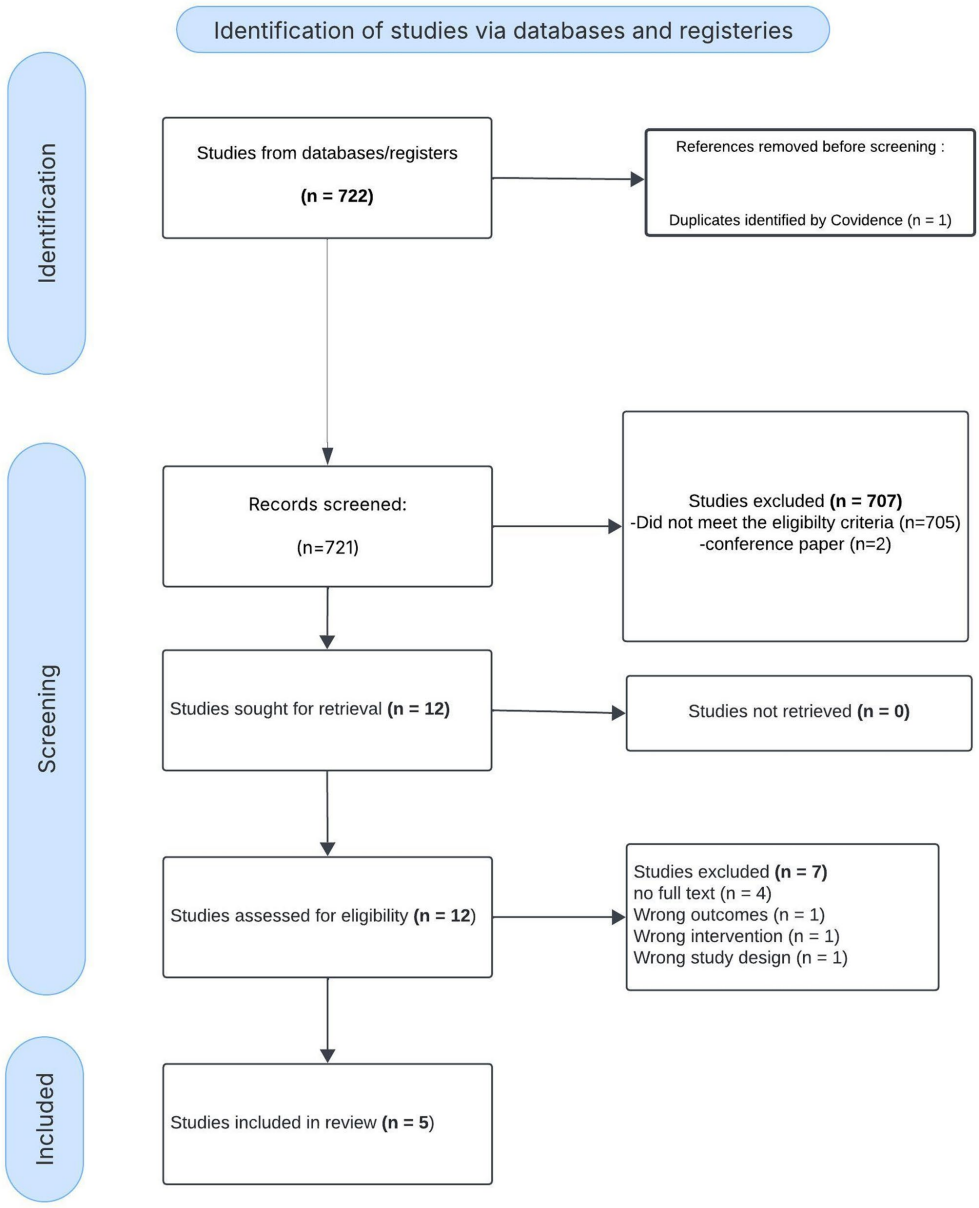
PRISMA Flow Diagram for Study Selection in the Systematic Review. This PRISMA flow diagram illustrates the study selection process. Out of 722 identified records, 721 were screened, with 707 excluded. After full-text assessment of 12 studies, 5 were included in the final review, while 7 were excluded due to ineligibility

Among 722 identified studies, 12 articles underwent full-text review, and 5 studies met all eligibility criteria and were included in the final analysis. Studies were excluded if they were non-original, did not compare chloride levels or did not report mortality or AKI. During the full-text screening process, 7 studies were excluded. four were removed due to the unavailability of full-text access, while the remaining three were excluded because their outcomes and result data did not align with the requirements of our subject or data needs. (Fig. 1). The inter-rater agreement for study inclusion was substantial, (κ = 0.83).

### Study characteristics

The five included studies evaluated 3,537 adults with cirrhosis. Among them, three were single-center retrospective cohort [19, 22, 26], one was a single-center prospective observational study [27], and one utilized the Medical Information Mart for Intensive Care III (MIMIC-III) database [20].

Regarding the definition of hypochloremia, three studies defined it as a serum chloride < 99 mEq/L [19, 20, 26], while one study used < 107.35 mmol/L [22]. The remaining study did not report specify an exact cutoff for hypochloremia but categorized patients into 2 groups: hypochloremia and normochloremia [27].

The etiology of cirrhosis varied across the included studies, reflecting a wide range of underlying causes. In Maiwall 2021 [27], alcohol-related liver disease was the leading cause, accounting for 61.5% of cases, followed by hepatitis B (7.9%), hepatitis C (8.9%), non-alcoholic steatohepatitis (NASH) (11.7%), cryptogenic causes (8.6%), and other etiologies (1.4%). Similarly, in J. Cheng 2023, hepatitis B virus was the predominant cause, responsible for 59.9% of cases, with hepatitis C virus contributing to 7.7%, alcohol-related liver disease to 8.8%, autoimmune liver disease to 4.4%, and other causes to 19.2%.

In Semmler 2023’s [26] ACLD cohort, alcohol-related liver disease accounted for 40% of cases, viral hepatitis for 35%, non-alcoholic fatty liver disease (NAFLD) for 7%, and other causes for 17%. Within the ICU cohort of Semmler 2023, alcohol-related liver disease remained the predominant etiology at 64%, followed by viral hepatitis (19%) and other causes (17%). Similarly, Sumarsono 2020 [19], identified alcohol-related liver as the most common etiology, contributing to 63% of cases, followed by viral hepatitis (39%), NASH (12%), and autoimmune liver disease (3%). Finally, in Yun Ji & Libin Li 2021, cirrhosis was attributed to alcohol-related causes in 48.8% of cases and non-alcoholic causes in 51.2%.

The reported comorbidities among patients with cirrhosis varied across the included studies. In Maiwall 2021 [27], 21.3% of patients had diabetes mellitus, 13.4% had arterial hypertension, and 75.2% had prior decompensation. Additionally, 79.4% experiencing at least one extrahepatic organ failure, 31.6% had two organ failures, and 13.4% having three organ failures. J. Cheng 2023 did not report specific comorbidities, focusing instead on hepatitis-related factors. In Semmler 2023’s [26] ACLD cohort, 72% of patients had varices, 62% had a history of or current decompensation, and 21% had a history of variceal bleeding. However, comorbidity data were not reported for Semmler 2023’s ICU cohort [26], Yun Ji & Libin Li 2021 [20], or Sumarsono 2020 [19].

**Fig. 2:**
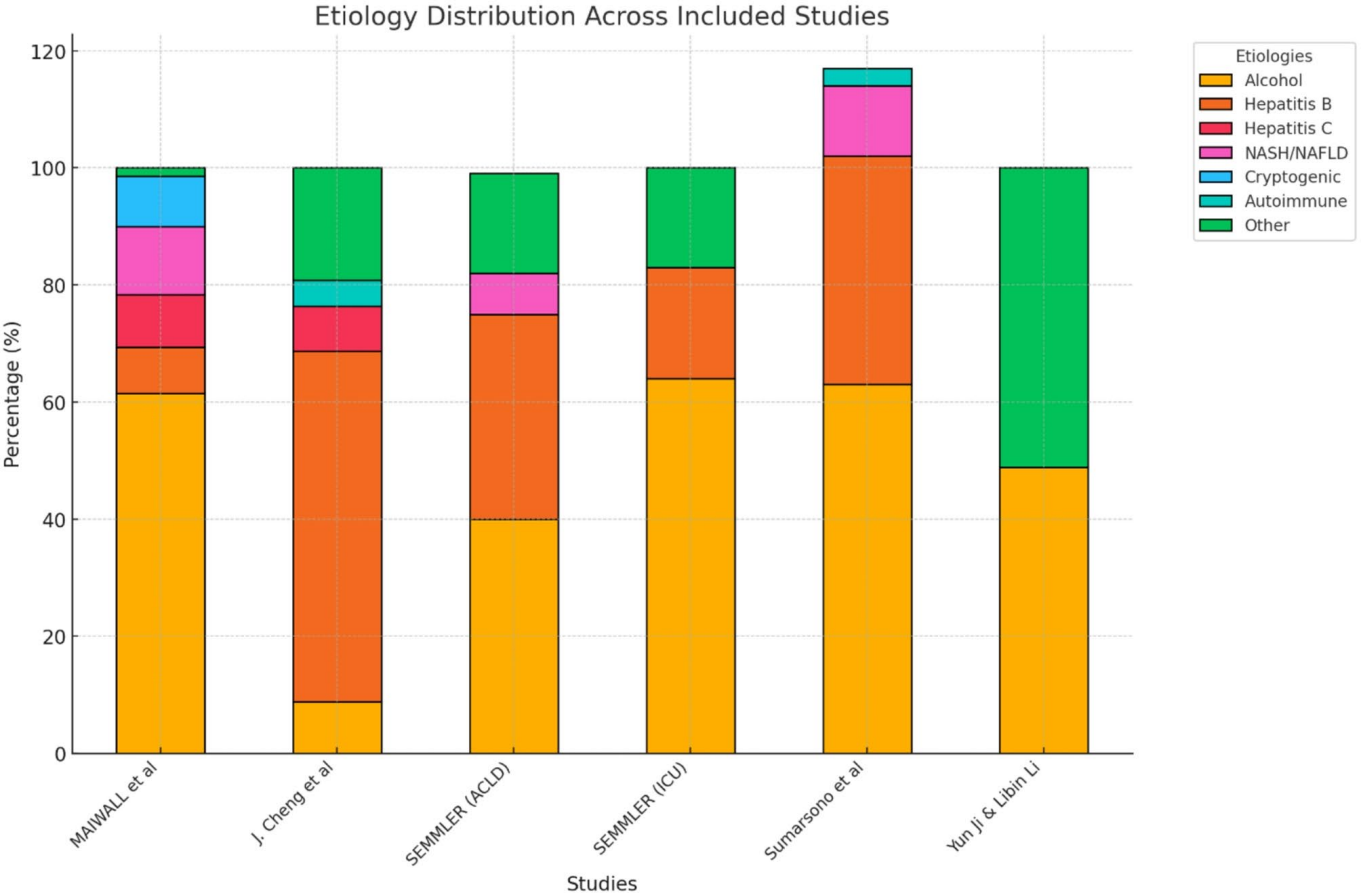
Etiology Distribution of Cirrhosis Across Included Studies. This bar chart illustrates the etiology distribution of cirrhosis across the included studies. The different colors represent various causes of cirrhosis, including alcohol-related liver disease, viral hepatitis (Hepatitis B and C), non-alcoholic steatohepatitis (NASH), cryptogenic causes, autoimmune liver disease, and other etiologies. The proportion of each etiology varies among studies, with alcohol being the predominant cause in most cohorts

**Fig. 3:**
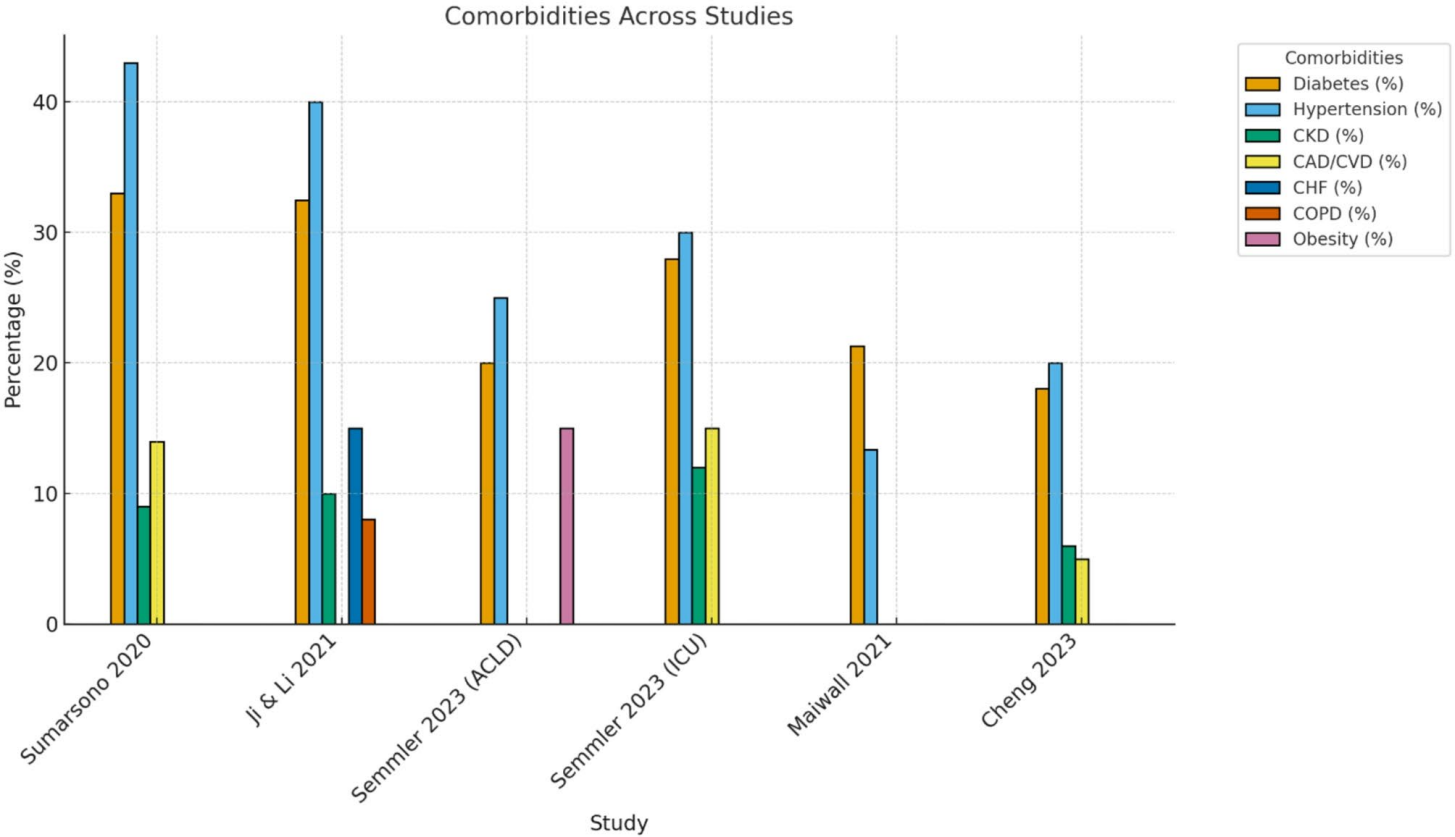
Reported Comorbidities in Patients with Cirrhosis Across Included Studies. Values represent the percentage of patients with reported cirrhosis-related complications in each study. Missing bars indicate that the variable was not reported. Semmler et al. presented separate ACLD and ICU cohorts, which are shown individually for clarity

**Table 2:** .Baseline characteristics of included studies^1^

First author, Year	Age	Males	Etiology of Cirrhosis	Comorbidities	Cirrhosis-related complications	MELD Score (mean ± SD)	SOFA Score (mean ± SD)	Hospitalization time (days)	Vasopressors, *n* (%)	Renal replacement therapy, *n* (%)
Maiwall. 2021	48 ± 11.4	252 (86.5)	Alcohol 61.5%/ Hepatis B 7.9% HEP C 8.9% NASH 11.7% Cryptogenic 8.6% Other 1.4%	Diabetes mellitus: 62 (21.3%) Arterial hypertension: 39(13.4%)	Prior decompensation: 219 patients (75.2%) At least one extrahepatic organ failure: 231 patients (79.4%) Two extra-hepatic organ failures: 92 patients (31.6%) Three extrahepatic organ failures: 39 patients (13.4%)	30.1 ± 8.4	11.6 ± 4.5	NR	NR	NR
J. Cheng. 2023	57.0 ± 10	115(63.2)	Hepatitis B virus 109(59.9) Hepatitis C virus 14(7.7) Alcohol 16(8.8) Autoimmune liver disease 8(4.4) Others 35(19.2)	Diabetes mellitus: 18% Hypertension: 20% Chronic kidney disease: 6% Coronary artery disease: 5%	All patients had portal hypertension with TIPS indication	10.6 ± 1.72/ MELD-Na 11.8 ± 1.5	NR	12 ± 1.5	NR	NR
Semmler. 2023, Cohort 1 (STABLE ACLD cohort)	54.3 ± 11.5	607 (68)	ALD 355 (40) Viral 316 (35) NAFLD 66 (7) Other154 (17)	Diabetes mellitus: 20% Hypertension: 25% Obesity: 15%	Varices: 72% History of or current decompensation: 62% History of variceal bleeding: 21%	11 ± 2 (9 to 17	NR	NR	NR	NR
Semmler. 2023, Cohort 2 (ICU cohort)	51.9 ± 11.9	130 (72)	ALD 116 (64) Viral 34 (19) Other 31 (17)	Diabetes mellitus: 28% Hypertension: 30% Chronic kidney disease: 12% Cardiovascular disease: 15%	Varices: 87% History of or current decompensation: 99% History of variceal bleeding: 31%	31 ± 4.25	NR	NR	NR	NR
Sumarsono. 2020	55 ± 0.5	270 (69)	Alcohol 243 (63) Viral 152 (39) Nonalcoholic steatohepatitis 46 (12) Autoimmune 10 (3)	Diabetes mellitus: 33% Hypertension: 43% Coronary artery disease: 14% Chronic kidney disease: 9%		MELD- Na 22 ± 3.75	8 ± 1.25	NR	109 (28)	43 (11)
Yun Ji & Libin Li. 2021	57 ± 11.31	270 (69)	Alcoholic: Total 593 (48.8) Non-alcoholic: Total 623 (51.2)	Diabetes mellitus: 30–35% Hypertension: 40% Congestive heart failure: 15% Chronic kidney disease: 10% COPD: 8%		20 ± 9.90	7 ± 4.24	NR	0	50 (4.1)

^1^This table summarizes baseline characteristics of patients included in the studies, including age, sex distribution, cirrhosis etiology, comorbidities, MELD and SOFA scores, hospitalization duration, use of vasopressors, and renal replacement therapy (RRT). Values are presented as mean ± standard deviation (SD) or number (percentage) where applicable. NR indicates data not reported in the respective study. Treatment details are presented as reported in the original studies. Information on renal replacement therapy, terlipressin, albumin, and other supportive measures was inconsistently available, and therefore could not be uniformly analyzed. Semmler et al. reported both a stable ACLD cohort and a critically ill ICU cohort, which are presented separately in this table. Other included studies primarily enrolled decompensated or critically ill patients

### Association of hypochloremia with mortality risk

**Fig. 4:**
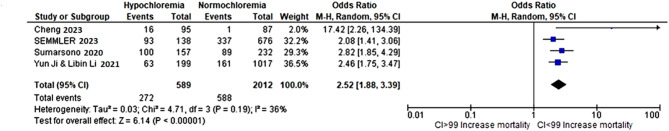
Forest Plot of the Association Between Hypochloremia and Mortality in Cirrhotic Patients. This forest plot shows the association between hypochloremia and mortality in cirrhotic patients, with an overall odds ratio (OR) of 2.52 [1.88, 3.39], indicating more than 2.5 times higher mortality risk in hypochloremic individuals. The pooled estimate (black diamond) is statistically significant (*p* < 0.00001), and heterogeneity is moderate (I² = 36%), suggesting consistency across studies

Among the included studies, four assessed the association between hypochloremia and all-cause mortality, encompassing a total of 2,601 patients, with 589 in the hypochloremia group and 2,012 in the normochloremia group. Our pooled analysis revealed a significant association between hypochloremia and increased all-cause mortality, with a combined odds ratio (OR) of 2.52 (95% CI: 1.88–3.39; I² = 36%, *p* < 0.0001;). Mortality rates in the hypochloremia group ranged from 16.8 to 53.8%, compared to 1.1–15.8% in the normochloremia group (Fig. 4).

### Correlation of chloride levels with creatinine and sodium

Among the included studies, the relationship between hypochloremia and creatinine levels was variably reported. Sumarsono 2020 [19] found that hypochloremia was associated with higher creatinine levels, reflecting worse renal function, with hypochloremic patients having significantly higher mean creatinine levels compared to those with normal chloride levels (1.7 mg/dL vs. 1.0 mg/dL, *p* < 0.01). Similarly, Ji and Li 2021 [20] observed significantly higher median creatinine levels in hypochloremic patients compared to those with normal chloride levels (1.4 mg/dL vs. 0.9 mg/dL, *p* < 0.001), further linking hypochloremia to impaired renal function. Cheng 2023 [22]. and Semmler 2023 [26]. did not report a direct relationship between chloride and creatinine levels. However, findings from Semmler 2023 change that change. suggest an indirect link between hypochloremia and renal dysfunction based on its overall impact on clinical outcomes.

Regarding the correlation between chloride and sodium levels, multiple studies consistently demonstrated a strong positive association. Cheng 2023 [22] reported a significant correlation between serum chloride and sodium levels (Spearman correlation coefficient: *r* = 0.451, *p* < 0.001). Sumarsono 2020 [19] identified a stronger correlation (*r* = 0.71, *p* < 0.0001), noting that hypochloremia frequently co-occurred with hyponatremia. Ji and Li found a similarly strong correlation (*r* = 0.771, *p* < 0.001) and highlighted that hypochloremia often coincided with hyponatremia (serum sodium < 135 mmol/L). Lastly, Semmler 2023 [26] reported significant positive correlations between sodium and chloride levels, with correlation coefficients of *r* = 0.634 in the ACLD cohort and *r* = 0.816 in the ICU cohort, underscoring the interdependence of these electrolytes in cirrhosis populations.

## Discussion

Our analysis suggests that hypochloremia is an independent predictor of mortality in patients with cirrhosis. Across the included studies, hypochloremic individuals had 152% higher odds of death compared with those with normal chloride levels. Although there was mild to moderate heterogeneity (I² = 36%), the association remained consistent across different study populations and is in line with previous work in heart failure and chronic kidney disease [14–16, 18, 28].These findings support the prognostic relevance of serum chloride as a marker distinct from sodium, reinforcing its role in outcome prediction in cirrhosis.

Beyond mortality, we also explored the relationship between chloride and kidney function. Several studies suggested potential associations with acute kidney injury (AKI) progression. For example, Maiwall 2021 [27] found that each 1 mmol/L increase in serum chloride during follow-up was associated with a 5% higher likelihood of progressive or persistent AKI, possibly related to chloride-rich fluid resuscitation. However, no consistent association was observed between baseline chloride levels and the development of AKI. Although chloride correlated with creatinine in some studies, its specific role in the pathophysiology of AKI in cirrhosis remains uncertain and requires further research.

The included studies varied in design, ranging from retrospective cohorts to prospective observational studies, with a combined sample of 3,537 patients. Most participants were middle-aged men (71%), and alcohol-related liver disease was the predominant etiology. While the overall quality of the studies was moderate to high, variability in methodology and in the definitions of hypochloremia limited the generalizability of the findings. Mechanistically, hypochloremia may contribute to adverse outcomes by reducing renal perfusion, activating the renin–angiotensin system, and promoting systemic vasoconstriction. These processes are particularly relevant in the pathophysiology of cirrhosis.

These findings highlight the importance of identifying chloride-depleted patients with cirrhosis and implementing strategies to prevent worsening depletion. Although this review does not yet support immediate changes to clinical practice, it provides a foundation for further work. Well-designed clinical trials comparing electrolyte-focused management with standard care are warranted to determine whether correction of hypochloremia can improve patient outcomes.

Strengths of this review include a comprehensive search strategy, adherence to PRISMA methodology, and systematic assessment of study quality. We focused on clinically meaningful outcomes such as mortality and AKI progression. Despite the inherent limitations of observational data, including retrospective design, inconsistent definitions of hypochloremia, and limited adjustment for confounders such as diuretic use, fluid balance, and disease severity, the findings were consistent. Five studies demonstrated a significant association between hypochloremia and mortality, strengthening confidence in this relationship. Although mild to moderate heterogeneity was observed (I² = 36%), this is unlikely to alter the conclusion that low chloride is strongly associated with poor outcomes in cirrhosis.

This review has several limitations. The number of eligible studies was small, and four older articles could not be retrieved in full text. Hospitalization and treatment details, including albumin and terlipressin therapy, renal replacement therapy, and other supportive measures, were inconsistently reported, which limited interpretation. Variability in disease severity, particularly in MELD and sodium levels, could not be fully addressed, and meta-regression was not feasible given the limited number of studies. All included studies assessed chloride at baseline on admission, which we clarified in the Results and tables, although the prognostic significance of changes during hospitalization remains uncertain. In Semmler’s work, the ICU and stable cohorts are now described separately. We did not assess publication bias with funnel plots or Egger’s test, as fewer than ten studies were available, in line with Cochrane guidance. Finally, only one study examined hypochloremia in predominantly compensated cirrhosis, while the others enrolled decompensated or critically ill patients. Because of this imbalance, a compensated-versus-decompensated subgroup analysis was not possible, underscoring the need for future studies to stratify outcomes by disease stage.

## Conclusions

Hypochloremia is strongly linked to increased mortality in adults with cirrhosis, although its relationship with renal injury remains unclear. Clinicians should closely monitor serum chloride levels when managing these patients. Our systematic review indicates that further research is necessary to elucidate the role of serum chloride in renal dysfunction and other organ failure in this population. Given the potential clinical repercussions, monitoring serum chloride levels in patients with cirrhosis may improve risk stratification and management. Further research, particularly prospective trials with standardized definition and protocols, is needed to develop interventions aimed at preventing and correcting dyschloremias to improve outcomes.

## Data Availability

All data generated or analyzed during this study are included in this published article and its supplementary information files. Additional datasets used and/or analyzed during the current study are available from the corresponding author on reasonable request.

## Abbreviations

AKI: Acute Kidney Injury
CKD: Chronic Kidney Disease
ICU: Intensive Care Unit
KDIGO: Kidney Disease Improving Global Outcomes
MELD: Model for End-Stage Liver Disease
MIMIC: Medical Information Mart for Intensive Care
NASH: Non-Alcoholic Steatohepatitis
NAFLD: Non-Alcoholic Fatty Liver Disease
OR: Odds Ratio
PRISMA: Preferred Reporting Items for Systematic Reviews and Meta-Analyses
PROSPERO: International Prospective Register of Systematic Reviews
ROBINS-I: Risk of Bias in Non-Randomized Studies of Interventions

## References

[1] Papper S. Fluid and electrolyte disturbances in cirrhosis. Am J Med Sci. 1976;272(1):53–6. 961715 10.1097/00000441-197607000-00006

[2] Lizaola B, Bonder A, Tapper EB, Mendez-Bocanegra A, Cardenas A. The changing role of sodium management in cirrhosis. Curr Treat Options Gastroenterol. 2016;14(2):274–84. 27037930 10.1007/s11938-016-0094-y

[3] John S, Thuluvath PJ. Hyponatremia in cirrhosis: pathophysiology and management. World J Gastroenterol. 2015;21(11):3197–205. 25805925 10.3748/wjg.v21.i11.3197 PMC4363748

[4] Gines P, Berl T, Bernardi M, Bichet DG, Hamon G, Jimenez W, et al. Hyponatremia in cirrhosis: from pathogenesis to treatment. Hepatology. 1998;28(3):851–64. 9731583 10.1002/hep.510280337

[5] Wood NL, VanDerwerken D, Segev DL, Gentry SE. Correcting the sex disparity in MELD-Na. Am J Transpl. 2021;21(10):3296–304. 10.1111/ajt.16731 PMC8500920 34174151

[6] Berend K, van Hulsteijn LH, Gans RO. Chloride: the queen of electrolytes? Eur J Intern Med. 2012;23(3):203–11. 22385875 10.1016/j.ejim.2011.11.013

[7] Moller S, Bendtsen F. The pathophysiology of arterial vasodilatation and hyperdynamic circulation in cirrhosis. Liver Int. 2018;38(4):570–80. 28921803 10.1111/liv.13589

[8] Shi HP, Deitch EA, Da Xu Z, Lu Q, Hauser CJ. Hypertonic saline improves intestinal mucosa barrier function and lung injury after trauma-hemorrhagic shock. Shock. 2002;17(6):496–501. 12069187 10.1097/00024382-200206000-00010

[9] Angle N, Hoyt DB, Coimbra R, Liu F, Herdon-Remelius C, Loomis W, et al. Hypertonic saline resuscitation diminishes lung injury by suppressing neutrophil activation after hemorrhagic shock. Shock. 1998;9(3):164–70. 9525322 10.1097/00024382-199803000-00002

[10] Chowdhury AH, Cox EF, Francis ST, Lobo DN. A randomized, controlled, double-blind crossover study on the effects of 1-L infusions of 6% hydroxyethyl starch suspended in 0.9% saline (voluven) and a balanced solution (Plasma volume Redibag) on blood volume, renal blood flow velocity, and renal cortical tissue perfusion in healthy volunteers. Ann Surg. 2014;259(5):881–7. 24253140 10.1097/SLA.0000000000000324

[11] Licata G, Di Pasquale P, Parrinello G, Cardinale A, Scandurra A, Follone G, et al. Effects of high-dose Furosemide and small-volume hypertonic saline solution infusion in comparison with a high dose of Furosemide as bolus in refractory congestive heart failure: long-term effects. Am Heart J. 2003;145(3):459–66. 12660669 10.1067/mhj.2003.166

[12] Shao M, Li G, Sarvottam K, Wang S, Thongprayoon C, Dong Y, et al. Dyschloremia is a risk factor for the development of acute kidney injury in critically ill patients. PLoS ONE. 2016;11(8):e0160322. 27490461 10.1371/journal.pone.0160322 PMC4974002

[13] Wu F, Lan Q, Yan L. Prognostic impact of serum chloride concentrations in acute heart failure patients: A systematic Rreview and meta-analysis. Am J Emerg Med. 2023;71:109–16. 37379618 10.1016/j.ajem.2023.05.035

[14] Zhang Y, Peng R, Li X, Yu J, Chen X, Zhou Z. Serum chloride as a novel marker for adding prognostic information of mortality in chronic heart failure. Clin Chim Acta. 2018;483:112–8. 29684381 10.1016/j.cca.2018.04.028

[15] Testani JM, Hanberg JS, Arroyo JP, Brisco MA, Ter Maaten JM, Wilson FP, et al. Hypochloraemia is strongly and independently associated with mortality in patients with chronic heart failure. Eur J Heart Fail. 2016;18(6):660–8. 26763893 10.1002/ejhf.477 PMC5471359

[16] Stankowski K, Villaschi A, Tartaglia F, Figliozzi S, Pini D, Chiarito M, et al. Prognostic value of hypochloremia on mortality in patients with heart failure: a systematic review and meta-analysis. J Cardiovasc Med (Hagerstown). 2024;25(7):499–510. 38809244 10.2459/JCM.0000000000001644

[17] Mandai S, Kanda E, Iimori S, Naito S, Noda Y, Kikuchi H, et al. Association of serum chloride level with mortality and cardiovascular events in chronic kidney disease: the CKD-ROUTE study. Clin Exp Nephrol. 2017;21(1):104–11. 27039905 10.1007/s10157-016-1261-0

[18] Kubota K, Sakaguchi Y, Hamano T, Oka T, Yamaguchi S, Shimada K, et al. Prognostic value of hypochloremia versus hyponatremia among patients with chronic kidney disease-a retrospective cohort study. Nephrol Dial Transpl. 2020;35(6):987–94. 10.1093/ndt/gfy299 30346587

[19] Sumarsono A, Wang J, Xie L, Chiang GC, Tielleman T, Messiah SE, et al. Prognostic value of hypochloremia in critically ill patients with decompensated cirrhosis. Crit Care Med. 2020;48(11):e1054–61. 32947468 10.1097/CCM.0000000000004620

[20] Ji Y, Li L. Lower serum chloride concentrations are associated with increased risk of mortality in critically ill cirrhotic patients: an analysis of the MIMIC-III database. BMC Gastroenterol. 2021;21(1):200. 33933032 10.1186/s12876-021-01797-3 PMC8088682

[21] Ferreira JP, Girerd N, Duarte K, Coiro S, McMurray JJ, Dargie HJ et al. Serum chloride and sodium interplay in patients with acute myocardial infarction and heart failure with reduced ejection fraction: an analysis from the high-risk myocardial infarction database initiative. Circ Heart Fail. 2017;10(2). 10.1161/CIRCHEARTFAILURE.116.003500 28159825

[22] Cheng J, Huang K, Mou JL, Lao YJ, Feng JH, Hu F, et al. Prognosis value of serum chloride on 1-year mortality in cirrhotic patients receiving transjugular intrahepatic portosystemic shunt. J Formos Med Assoc. 2023;122(9):911–21. 36878767 10.1016/j.jfma.2023.02.009

[23] Grodin JL, Verbrugge FH, Ellis SG, Mullens W, Testani JM, Tang WH. Importance of abnormal chloride homeostasis in stable chronic heart failure. Circ Heart Fail. 2016;9(1):e002453. 26721916 10.1161/CIRCHEARTFAILURE.115.002453 PMC4702267

[24] Covidence. Better systematic review management 2024 [January 24, 2025]. Available from: https://www.covidence.org/

[25] Sterne JA, Hernan MA, Reeves BC, Savovic J, Berkman ND, Viswanathan M, et al. ROBINS-I: a tool for assessing risk of bias in non-randomised studies of interventions. BMJ. 2016;355:i4919. 27733354 10.1136/bmj.i4919 PMC5062054

[26] Semmler G, Scheiner B, Balcar L, Paternostro R, Simbrunner B, Pinter M, et al. Disturbances in sodium and chloride homeostasis predict outcome in stable and critically ill patients with cirrhosis. Aliment Pharmacol Ther. 2023;58(1):71–9. 37016513 10.1111/apt.17507

[27] Maiwall R, Pasupuleti SSR, Chandel SS, Narayan A, Jain P, Mitra LG, et al. Co-orchestration of acute kidney injury and non-kidney organ failures in critically ill patients with cirrhosis. Liver Int. 2021;41(6):1358–69. 33534915 10.1111/liv.14809

[28] Grodin JL, Simon J, Hachamovitch R, Wu Y, Jackson G, Halkar M, et al. Prognostic role of serum chloride levels in acute decompensated heart failure. J Am Coll Cardiol. 2015;66(6):659–66. 26248993 10.1016/j.jacc.2015.06.007

